# Non-Sexual Transmission of *Trichomonas vaginalis* in Adolescent Girls Attending School in Ndola, Zambia

**DOI:** 10.1371/journal.pone.0016310

**Published:** 2011-01-31

**Authors:** Tania Crucitti, Vicky Jespers, Chanda Mulenga, Shepherd Khondowe, Judith Vandepitte, Anne Buvé

**Affiliations:** 1 HIV/STI Reference Laboratory, Institute of Tropical Medicine, Antwerp, Belgium; 2 HIV/STI Epidemiology and Control Unit, Institute of Tropical Medicine, Antwerp, Belgium; 3 Tropical Diseases Research Centre, Ndola, Zambia; 4 HIV/STI Epidemiology and Control Unit, Institute of Tropical Medicine, Antwerp, Belgium; University of Cape Town, South Africa

## Abstract

**Objectives:**

To identify risk factors for trichomoniasis among young women in Ndola, Zambia.

**Method:**

The study was a cross-sectional study among adolescent girls aged 13-16 years in Ndola, Zambia. Study participants were recruited from schools in selected administrative areas that represented the different socio-economic strata in town. Consenting participants were interviewed about their socio-demographic characteristics; sexual behaviour; and hygiene practices. Self-administered vaginal swabs were tested for *Trichomonas vaginalis*. HSV-2 antibodies were determined on serum to validate the self-reported sexual activity.

**Results:**

A total of 460 girls participated in the study. The overall prevalence of trichomoniasis was 27.1%, 33.9% among girls who reported that they had ever had sex and 24.7% among virgins. In multivariate analysis the only statistically significant risk factor for trichomoniasis was inconsistent use of soap. For the virgins, none of the risk factors was significantly associated with trichomoniasis, but the association with use of soap (not always versus always) and type of toilet used (pit latrine/bush versus flush toilet) was of borderline significance.

**Conclusion:**

We found a high prevalence of trichomoniasis in girls in Ndola who reported that they had never had sex. We postulate that the high prevalence of trichomoniasis in virgins in Ndola is due to non-sexual transmission of trichomoniasis via shared bathing water and inconsistent use of soap.

## Introduction

*T. vaginalis* is a flagellated eukaryotic organism responsible for the commonest curable sexually transmitted infection worldwide with nearly 170 million new cases each year [1]. Infection with *T. vaginalis* may present as an asymptomatic infection or an inflammatory disease. The parasite may cause vaginitis, cervicitis, and urethritis [2].The infection has been associated with pelvic inflammatory disease, adverse pregnancy outcome, and increased risk of acquisition and transmission of HIV infection [3]–[5].

At a symposium on Trichomonas infections in Rheims in 1957, the majority of delegates agreed that trichomoniasis was usually transmitted by sexual intercourse and that it should be regarded as a venereal disease [6].There is no doubt that sexual transmission predominates and is the most important mode of transmission of trichomoniasis. However, there are several reports of non-sexual transmission. Adu-Sarkodie reported probable transmission of *T. vaginalis* within a family in Ghana and hypothesized that transmission occurred from the mother to her children through the sharing of bathing implements [7]. Charles described an epidemic of trichomoniasis in young girls in a rural area in India. He suggested that transmission had occurred through contaminated water [8]. *T. vaginalis* can indeed survive outside the human body in a wet environment for more than three hours [9]–[11].

In 1997–1998 a population-based study was conducted in four African cities, two in West Africa (Cotonou in Benin and Yaoundé in Cameroon) and two in East Africa (Kisumu in Kenya and Ndola in Zambia) to identify factors that could explain the differences in the rate of spread of HIV between different regions in sub-Saharan Africa [12]. The prevalence of *T. vaginalis* infection was much higher in the two East African cities [13]. The most striking finding however was the high prevalence of trichomoniasis in women in Ndola (Zambia) who denied that they had ever had sexual intercourse. The prevalence of trichomoniasis was 40% among women who denied sexual activity, whereas in the other cities the prevalence ranged between 2.9% and 7.7%. We hypothesized that in Ndola non-sexual transmission of *T. vaginalis* is very frequent.

We decided to explore this issue and to conduct further research on the epidemiology of trichomoniasis in women in Ndola. This paper presents the results of the study which we conducted among young girls aged 13 to 16 years. The objective of this study was to identify risk factors for trichomoniasis among young women in Ndola.

## Methods

### Ethical Committee

The study was approved by the Ethics Committees of the Tropical Diseases Research Centre in Ndola and the Institute of Tropical Medicine in Antwerp.

### Study Design and Sample

We set out to compare the prevalence of trichomoniasis in young girls who were sexually active and young girls who were not sexually active, and took the age range of 13 to 16 years for our study population. Study participants were recruited from schools where permission was obtained from the headmaster and teaching staff. A meeting was then organized with research staff and girls aged 13–16 years during which the study was explained. Girls who were interested in participating in the study were given an appointment for the informed consent procedure. Informed consent for an interview and the collection of specimens was first sought from the parents or guardians. Girls could participate in the study only when written consent was obtained from the parents or guardian and assent from the girls themselves.

Assenting study participants were interviewed face-to-face by a trained interviewer about their socio-demographic characteristics, sexual behaviour, and hygiene practices. The girls were asked to submit two self-collected swabs: a cotton swab that was immediately inoculated in a culture medium (InPouch, Biomed Diagnostics, San Jose, California) and kept at room temperature, and a BD BBL CultureSwab EZ (Becton Dickinson BBL, Maryland, MD, USA) which was kept in a cool box. A study nurse took a 10cc blood sample which was kept at room temperature. All specimens were transported to the laboratory within seven hours of collection.

The face-to-face interview and specimen collection were carried out either at the home of the girl or at the school and in the absence of parents, guardians or teachers. The girls received an appointment at the Tropical Diseases Research Centre clinic (TDRC), where they could collect their results of the trichomoniasis test and receive treatment free of charge.

### Laboratory procedures

Upon arrival at the laboratory of the TDRC, the BD BBL CultureSwabs EZ were stored at −20°C until shipment. InPouch culture media were incubated at 37°C and examined for the characteristic trichomonad morphology and motility up to seven days after inoculation. After final reading, the pouches were stored at −80°C until shipment.

The blood specimens were centrifuged and the sera were kept at −20°C at the TDRC, until shipment. All specimens were shipped on dry ice to the Institute of Tropical Medicine in Antwerp (ITM), Belgium.

At the ITM the vaginal swab specimens were tested for *T. vaginalis* by polymerase chain reaction (PCR). After thawing the swabs at room temperature for 30 minutes, 1250 µl diluted PBS [pH 7.4] were added to the swab and gently vortexed for at least 15 seconds. Aliquots of 250 µl were extracted using the QIAamp® DNA minikit (Qiagen, Hilden, Germany), following the manufacturer's instructions. The extracted DNA was eluted with 250 µl of Tris-acetate-EDTA -buffer [pH 7.4]. For the amplification of *T. vaginalis* we used TVK3/7 primers, and positive results were confirmed using IP1/IP2 primers [14], [15]. Specimens that tested positive on the two different primer sets were considered to be true positive.

The *T. vaginalis* amplicons were detected using an enzyme immuno assay (EIA). The PCR assays were performed as described elsewhere [16].

The β_2_ microglobulin gene amplification was carried out on each vaginal specimen to control for PCR inhibition [16]. In order to validate the InPouch microscopical readings, 500 µl of all InPouch media were extracted and tested for *T. vaginalis* by PCR as described above.

The serum specimens were tested for antibodies against HSV-2 (Kalon Biological Ltd, Surrey, UK), following the manufacturer's instructions.

### Data analysis

Study participants were considered to be infected with *T. vaginalis* if the InPouch culture was positive, or if the PCR result on the InPouch or on the swab was positive, using the two different primer sets. Study participants were considered to be virgins if they reported that they had never had sexual intercourse and if the serum sample was negative for antibodies against HSV-2. Girls who denied that they had ever had sexual intercourse and were positive for HSV-2 infection were excluded from the risk factor analysis.

The following risk factors for trichomoniasis were explored in bivariate analysis: age; socio-economic status of the area of residence; bathing facilities; use of soap when bathing; sharing of bathing implements; type of toilet; sharing of bed; sexual activity; HSV-2 infection. Factors that were significantly associated with trichomoniasis at the 0.1 level, as well as age, were included in a multivariate regression model. All data analyses were done using SPSS for Windows version 15.0.

## Results

A total of 460 girls aged 13–16 years participated in the study. We could establish the presence or absence of *T. vaginalis* infection in 439 girls. Fifteen girls did not submit a sample and samples of another 6 girls gave inconclusive results due to PCR inhibition. InPouches of 5 girls could not be retrieved.

Table 1 describes the socio-economic characteristics, hygiene practices and sexual behaviour of the 460 girls. Twenty three percent of the girls lived in a poor or very poor area of town. Very poor areas of town lacked running water or electricity, poor areas of town had running water and pit latrines but the general socio-economic status of the residents was low. Nearly all girls took a bath or washed their body daily, and the majority (75%) used a basin. Soap was always used for washing by 70%, and 45% of the girls used soap inside the vagina. More than half of the girls used a flush toilet. Seventy-three percent of the girls reported that they shared their bedding with someone else, most often a sister or the mother. There was no significant difference in the proportion of girls who shared their bed according to whether they slept in a bed, on a mattress or on a mat (data not shown).

**Table 1:** Characteristics of the study population.

		N = 460	%
**Socio-economic status**			
Age	13	164	35.7
	14	111	24.1
	15	82	17.8
	16	103	22.4
Religion	Christian	450	98.0
	Other	9	2.0
Earning an income	Yes	28	6.1
	No	431	93.9
Area of residence	Better off	353	76.7
	Very poor/poor	107	23.3
**Hygiene practices and sleeping arrangements**			
Facilities for washing	Tub	58	12.6
	Shower	58	12.6
	Basin	343	74.6
	Other	1	0.2
Frequency of bathing or washing the whole body	Daily	449	97.6
	Less than daily	11	2.4
Use of soap when washing	Always	323	70.2
	Often	111	24.1
	Rarely	26	5.7
Uses soap inside the vagina	Yes	206	44.8
	No	254	55.2
Shares towels	Yes	245	53.3
	No	210	46.2
Type of toilet used	Flush toilet	255	55.4
	Private pit latrine	155	33.7
	Shared pit latrine	46	10.0
	Bush	4	0.9
Sleeps on…	Bed	232	50.4
	Mattress	115	25.0
	Mat	94	20.4
	Other	19	4.1
Shares the bed, mattress …	Yes	336	73.0
	No	124	27.0
**Sexual activity and reproductive health**			
Has started menstruating	No	154	33.6
	Yes	304	66.4
Sexual intercourse reported	No	397	86.3
	Yes	63	13.7
Sexual activity reported or HSV-2 positive	No	380	82.6
	Yes	80	17.4
Number of lifetime sex partners (reported by those who reported sexual activity)	1	43	72.9
	2	9	15.3
	3	3	5.0
	4	4	6.8
Abnormal vaginal discharge	No	410	89.7
	Yes	47	10.3

Not all numbers tally due to missing information.

Sixty-three girls (13.7%) reported that they had ever had sexual intercourse with the majority (73%) reporting 1 lifetime sex partner. Of the 397 girls who denied that they had ever had sex, 17 (4%) had HSV-2 antibodies, suggesting initiation of sexual activity. If we include these 17 girls in the group of ‘ever had sex’ the total percentage of girls who ever had sex was 17.4%.

The overall HSV-2 prevalence among the adolescent girls in this study was 5.5% (25/450).

Of the 439 samples available for analysis, 79 were positive on InPouch, 37 were positive on PCR on the swab but negative on the InPouch and 3 were positive on the PCR on the InPouch. The overall prevalence of *T. vaginalis* infection was 27.1% (119/439). Fourteen (11.7%) of the 119 girls with *T. vaginalis* infection complained of vaginal discharge and 10 (8.4%) complained of a bad smell and/or dysuria. The prevalence of complaints was not significantly different among girls with or without trichomoniasis (29.8% vs. 26.7%). Complaints were also not more frequent among girls who had a positive InPouch compared to girls who were only positive on PCR.

Table 2 presents risk factors for *T. vaginalis* infection. In univariate analysis trichomoniasis was associated with taking a bath in a basin as opposed to using a shower or a bath tub. Girls who used soap all the time were less likely to have trichomoniasis. Furthermore trichomoniasis was associated with using a pit latrine or the bush as opposed to a flush toilet. Among the girls who reported that they had ever had sex prevalence of trichomoniasis was 33.9% and among the virgins it was 24.7%. This difference was of borderline significance. Among the girls who reported they had ever had sex *T. vaginalis* infection was not associated with higher numbers of lifetime sex partners but trichomoniasis was associated with HSV-2 infection. In multivariate analysis the only statistically significant risk factor for *T. vaginalis* was inconsistent use of soap. Sexual activity was not significantly associated with trichomoniasis and the association with HSV-2 infection was of borderline significance. Among the virgins a borderline association of *T. vaginalis* infection with infrequent use of soap, OR 1.6 (95% CI: 0.9 – 2.7) and type of toilet, OR 1.6 (95% CI: 0.9 – 2.7) was present.

**Table 2:** Risk factors for *T. vaginalis* infection.

N = 439		% *T. vaginalis*	OR	Unadjusted P value	Adjusted OR	Adjusted P value
Age	13	23.9% (38/159)	1	0.6	1	0.2
	14	26.6% (29/109)	1.2 (0.7–2.0)		1.2 (0.7–2.3)	
	15	29.7% (22/74)	1.3 (0.7–2.5)		1.6 (0.9–3.2)	
	16	30.9% (30/97)	1.4 (0.8–2.5)		1.5 (0.8–2.9)	
Area of residence^1^	Better off	23.5% (66/281)	1	0.6		
	Poor/very poor	28.9% (24/83)	1.3 (0.8–2.1)			
Facilities for washing	Tub/shower	18.0% (20/111)	1	0.01	1	0.11
	Basin/other	30.2% (99/328)	2.0 (1.1–3.4)		1.6 (0.9–2.9)	
Use of soap when washing	Always	24.3% (75/309)	1	0.04	1	0.07
	Often/rarely	33.8% (44/130)	1.6 (1.0–2.5)		1.6 (1.0–2.6)	
Uses soap inside the vagina	Yes	27.3% (54/198)	1	0.9		
	No	27.0% (65/241)	1.0 (0.7–1.6)			
Shares towels^1^	No	28.7% (58/202)	1	0.6		
	Yes	26.3% (61/232)	0.9 (0.6–1.4)			
Type of toilet used	Flush toilet	22.4% (54/241)	1	0.02	1	0.30
	Pit latrine/bush	32.8% (65/198)	1.7 (1.1–2.6)		1.3 (0.8–2.1)	
Sleeps on…	Bed/mattress	25.0% (82/328)	1	0.09	1	0.27
	Mat/other	33.3% (37/111)	1.5 (0.9–2.4)		1.4 (0.8–2.3)	
Shares the bed, mattress …	No	29.8% (36/121)	1	0.4		
	Yes	26.1% (83/318)	0.8 (0.5–1.3)			
Has started menstruating^1^	No	26.5% (40/151)	1	0.8		
	Yes	27.6% (79/286)	1.1 (0.7–1.7)			
Ever had sex*	No	24.7% (90/364)	1	0.14	1	0.9
	Yes	33.9% (20/59)	1.6 (0.9–2.8)		1.1 (0.5–2.1)	
Number of lifetime sex partners^1^	0	24.7% (90/364)	1	0.5		
	1	33.3% (13/39)	1.5 (0.8–3.1)			
	>1	31.3% (5/16)	1.4 (0.5–4.1)			
HSV-2 positive^1^	No	25.1% (102/407)	1	0.004	1	0.06
	Yes	58.3% (14/24)	4.2 (1.8–9.7)		4.7 (0.9–23.4)	

*Girls who denied that they had ever had sex and were HSV-2 positive were excluded.

1 The total differs from 439 because of missing data for this factor.

## Discussion

This study confirms our previous finding of a high prevalence of trichomoniasis in women in Ndola who report that they had never had sex [13]. In the study of 1996–1997 we found an overall prevalence of 40% among women aged 15–49 years who never had sexual intercourse [13]. Among women who denied sexual activity and were HSV-2 negative, the prevalence of trichomoniasis was 36.4% (24/66) in the adult group and 44.8% (13/29) among girls aged 15-16 years (A. Buvé, personal communication). In the present study we found a lower prevalence among girls who reported they had never had sex, 24.7% in the age group 13–16 years and 26.6% in the age group 15–16 years. The lower prevalence of trichomoniasis in this study can be explained by the fact that all the girls in this study were attending school, whereas in the previous study women were recruited from the general population and were probably on average of lower socio-economic status.

It is well known that self reported data on sexual behaviour are not reliable. The first possible explanation for our finding is that young girls have underreported their sexual activity. However it is highly unlikely that this is the only explanation for the high prevalence of trichomoniasis in girls who stated that they had never had sex. The prevalence of trichomoniasis in girls who reported that they had initiated sexual activity was 33.9%. If we assume that trichomoniasis is only transmitted through sexual intercourse, then, in order to find a prevalence of 24.7% among girls who said they had never had sex, we would have to accept that 78% of all girls aged 13 to 16 years in Ndola ever had sex and that the median age at first sexual intercourse is well below 13 years. This is rather unlikely and in contrast with the results of recent surveys on sexual behaviour in Zambia. In those surveys 14% of young women in urban areas reported that they had their first sexual experience before the age of 15 years, and the median age at first sexual intercourse was 17 years (http://www.measuredhs.com/hivdata/surveys) [17].

Buvé *et al.* suggested that trichomoniasis in virgins might be due to colonisation of the vagina by the intestinal parasite *Pentatrichomonas. hominis*, which is very difficult to distinguish from *T. vaginalis* on microscopic examination [13]. We rejected this hypothesis in a previous publication and confirmed the earlier findings of a study conducted by Adu-Sarkodie *et al.* in Ghana [18], [19]. The authors concluded that in their study population *P. hominis* played no role in vaginal trichomoniasis.

We postulate that the high prevalence of trichomonas infection in young girls in Ndola who reported to be virgins, is due to frequent non-sexual transmission of trichomoniasis. In the analysis of risk factors, the only factor that was statistically significantly associated with trichomoniasis, was infrequent use of soap. In addition the risk of trichomoniasis was increased in girls who used a basin for washing but this association was only of borderline significance. Risk of trichomoniasis was also increased, but not significantly, in girls who used a pit latrine or the bush as opposed to a flush toilet and in girls who slept on a mat instead of a bed or mattress. These results suggest that transmission of *T. vaginalis* may occur during bathing. We hypothesise that bathing in water that is shared with other women in the household is a route of non-sexual transmission of *T. vaginalis* in young girls. Another possible route of transmission may be manipulation of the genitals of the young girls by an older woman or by other girls who are themselves infected. Elongation of the labia is traditionally practised by people in Zambia as part of preparations for marriage. Unfortunately we did not collect any data on this practice. But a case of transmission of *T. vaginalis* following manipulation of the genitals by a traditional healer has recently been reported from the Gambia [20].

Although trichomoniasis is very prevalent worldwide, there is still some uncertainty about its significance in terms of morbidity and mortality [21]. Several studies have found trichomoniasis to be associated with an increased risk of acquiring HIV infection [5], [22]. A meta-analysis published in 2001 found an effect of trichomoniasis on HIV acquisition of 1.5 (95%CI: 1.2–1.9) [23]. A recent publication of a nested-case control study demonstrated a strong association between vaginal trichomoniasis and subsequent acquisition of HIV infection (adjusted OR, 2.74; 95% CI, 1.25–6.00) [24]. There is also mounting evidence that infection with *T. vaginalis* increases the risk of onward transmission of HIV. In Malawi HIV infected men with urethritis due to *T. vaginalis* were found to have an increased concentration of HIV virus particles in the semen [25]. A more recent study showed that treatment of trichomoniasis was able to reduce the HIV-1 RNA vaginal levels, confirming the findings of a previous study [26], [27]. However, more research is needed on the role of *T. vaginalis* infection in acquisition and transmission of HIV. In the study in Ndola conducted in 1996–1997 we found that 16% of girls in the general population aged 15–19 years were HIV infected [12]. The reasons for this high prevalence of HIV infection in young girls are not entirely clear. HSV-2 infection may play an important role in increasing the vulnerability of young girls for HIV, but we can not exclude trichomoniasis as a risk factor in these girls. These observations call for better screening for *T. vaginalis* and treatment, especially in young and vulnerable women.

In conclusion, we confirmed the high prevalence rate of *T. vaginalis* among young girls in Ndola who denied that they had ever had sex. We have not been able to identify the routes of transmission and further studies are needed to improve our understanding of the routes of transmission of *T. vaginalis*.
